# Motivational consequences of counterfactual mindsets: Does counterfactual structure influence the use of conservative or risky tactics?

**DOI:** 10.1007/s11031-022-09979-6

**Published:** 2022-09-10

**Authors:** Kevin Winter, Kai Epstude

**Affiliations:** 1 Leibniz-Institut für Wissensmedien, Schleichstraße 6, D-72076 Tübingen, Germany; 2 University of Groningen, Groningen, The Netherlands

**Keywords:** Counterfactual mindsets, Regulatory focus, Tactics in decision-making, Motivational states

## Abstract

Motivational states are important determinants of human behavior. Regulatory focus theory suggests that a promotion focus stimulates risky behavior, whereas a prevention focus fosters conservative tactics. Previous research linked counterfactual structure with regulatory focus. Extending this work, we predicted that additive counterfactual mindsets (“If only I had…”) instigate risky tactics in subsequent situations, whereas subtractive counterfactual mindsets (“If only I had NOT…”) lead to conservative tactics. We tested this prediction and the underlying assumptions in four preregistered studies (total *N* = 803) and obtained consistent null results. Additive and subtractive counterfactual mindsets did not elicit different tactics – neither on behavioral nor on self-report measures – and they did not influence participants’ motivation compared to a neutral control condition. Likewise, our results put doubts on previous findings on counterfactuals and regulatory focus as well as regulatory focus and conservative or risky behavior. More general implications for research on counterfactuals and motivation are discussed.

## Introduction

Motivational states are key drivers of our everyday behavior. Imagine, for instance, you are entering salary negotiations for your new job. What are the tactics you are likely to apply in such a situation? Would you be bold to get the best outcome for yourself or would you play safe to not spoil the relation with your new boss? This decision probably depends on the motivational state you are in, for instance, whether you are in a promotion focus striving for accomplishments and aspirations or in a prevention focus concerned with safety and responsibilities. These different motivational states or self-regulatory foci are associated with strategic concerns of vigilance and eagerness (Higgins, 1997) that might translate into the application of conservative or risky tactics at the behavioral level (Scholer et al., 2008). But how can one or the other type of tactics be activated?

The way we deal with the antecedents of past failure influences our motivation in future situations. Previous research has, for instance, linked regulatory focus to the occurrence of specific mental operations in response to failure. Failures of commission elicit subtractive counterfactuals (“If only I had not…”) that activate a prevention focus, whereas failures of omission provoke additive counterfactuals (“If only I had…”) that are related to promotion focus (Roese et al., 1999). To date, however, there is no research demonstrating that subtractive or additive counterfactuals instigate different (i.e., conservative vs. risky) tactics in a subsequent task.

The current research aims at extending the previous findings on the motivational effects of counterfactual thoughts and tests their impact on the choice of conservative or risky tactics in decision-making. We examine whether different types of counterfactual thoughts produce broader behavioral changes.

## The consequences of counterfactual thinking

Counterfactual thoughts are mental simulations about alternative outcomes to past events and frequently occur in our everyday reasoning (Epstude & Roese, 2008; Kahneman & Miller, 1986; Markman & McMullen, 2003; Roese & Epstude, 2017). Individuals reflect on their past mishaps, and form intentions for future actions. For instance, thinking about how the outcome of a financial investment or a study exam could have been worse, influences the motivation to change one’s behavior in future investments or exams. In these situations, counterfactuals increase the motivation to change, if they elicit negative affect but do so less when provoking positive affect (McMullen & Markman, 2000). These studies show that counterfactuals can have context-specific effects on motivation. That is, they impact motivation in contexts that are related to the situation that caused the counterfactuals in the first place.

Besides affecting behavior related to the content of a specific thought, counterfactuals can also have broader consequences independent of the thought content. One way by which these content-neutral effects were previously demonstrated is the induction of a so-called counterfactual mindset (Galinsky & Moskowitz, 2000). Such a mindset affects performance and behavior in unrelated situations in diverse ways. For instance, the induction of a counterfactual mindset has been shown to alter decision-making in subsequent group settings (Ditrich et al., 2019; Kray & Galinsky, 2003; Liljenquist et al., 2004), performance in unrelated cognitive tasks (Kray et al., 2006; Markman et al., 2007), and judgements about outgroups (Winter et al., 2022). Only little research has, however, dealt with the broader motivational consequences of counterfactual mindsets that are especially relevant to the adaption of future behavior.

The literature distinguishes between counterfactuals that involve mentally subtracting or mentally adding elements from or to a factual situation (i.e., counterfactual structure; Roese 1994). Subtractive counterfactuals (“If only I had not…”) usually arise from failures of action, whereas failures of inaction rather lead to additive counterfactuals (“If only I had…”). It is plausible that specific types of failures and the resulting (counterfactual) thoughts elicit specific strategies to adapt future behavior. But how might the motivational consequences of counterfactual mindsets look like?

## Counterfactual structure and self-regulation

Regulatory focus theory differentiates between a promotion focus in which people regulate nurturance needs and a prevention focus in which people regulate security needs (Higgins, 1997). These different motivational states are assumed to result in different behavioral strategies. When in a promotion focus, people should employ eager strategies in order to achieve gains and omit non-gains. When in a prevention focus, people should employ vigilant strategies in order to avoid losses and ensure non-losses. That these strategies transfer to actual behavior has been empirically demonstrated across domains as diverse as political decisions (Boldero & Higgins, 2011), cognitive task performance (Crowe & Higgins, 1997; Friedman & Förster, 2001), or car driving behavior (Hamstra et al., 2011).

The link between regulatory focus and behavior has, however, been revisited since. More recent research proposes a differentiation between strategies and tactics as a consequence of regulatory focus (Scholer et al., 2008). While strategies are general behavioral orientations, tactics refer to the actual behavior that is performed in a concrete situation (e.g., making conservative or risky decisions). On a strategic level, prevention focus is linked to vigilance and promotion focus to eagerness. But the consequences for actual behavior (i.e., the tactical means) are not as straightforward as suggested by earlier research. Indeed, both promotion and prevention focus can lead to the adoption of either conservative or risky tactics if the respective behavior serves the underlying goal (Scholer et al., 2010; Zou et al., 2014). Still, the “classical” pattern of promotion focus leading to more risky and prevention focus to more conservative tactics should occur when the context does not provide clear information on the possibilities of goal-pursuit (e.g., in a neutral recognition task; Crowe & Higgins 1997; Friedman & Förster, 2001; see also Scholer et al., 2008). Given the recent developments in research on regulatory focus, however, it seems necessary to revisit the relationship between regulatory focus and conservative or risky tactics under neutral conditions.

Previous theorizing connects subtractive counterfactuals with prevention focus and additive counterfactuals with promotion focus (Epstude & Roese, 2008; Roese & Epstude, 2017). This assumption rests on the notion that counterfactuals are goal-related and that specific thoughts about the antecedents of an event might increase the desirability of specific end states (Roese et al., 1999). Accordingly, subtractive counterfactuals should lead to a prevention-focused motivational state as they are resulting from failures of action. On the contrary, additive counterfactuals that arise in response to failures of inaction should increase promotion focus. So far, however, the empirical underpinning of this relationship is scarce and mostly relies on correlational findings (Roese et al., 1999, 2006). To the best of our knowledge, there is only one published piece of work that directly tests the causal effect of counterfactual thinking on regulatory focus. In one experiment, an induced counterfactual mindset led to different effects on regulatory focus depending on counterfactual structure (Roese et al., 1999; Experiment 2). Participants who generated subtractive counterfactuals later found prevention related goals more important (e.g., “not making enemies”), whereas generating additive counterfactuals increased the importance of promotion related goals (e.g., “making friends”). Still, it is unclear whether the importance attributed to certain goals translates into behavioral intentions or actual behavior.

The current research aims to answer the question whether different counterfactual mindsets would lead to the use of different tactics in decision-making. We base our predictions on two assumptions derived from the literature: (1) that subtractive (additive) counterfactuals lead to a prevention (promotion) focus (Roese et al., 1999, 2006), and that (2) these motivational states translate into distinct behavioral tendencies with a prevention (promotion) focus resulting in more conservative (risky) tactics (Boldero & Higgins, 2011; Crowe & Higgins, 1997; Friedman & Förster, 2001; Hamstra et al., 2011). Against this theoretical background, we hypothesize that:

### H1

A subtractive (vs. additive) counterfactual mindset should lead to the use of more conservative and less risky tactics.

## Overview of the current research

**Fig. 1:**
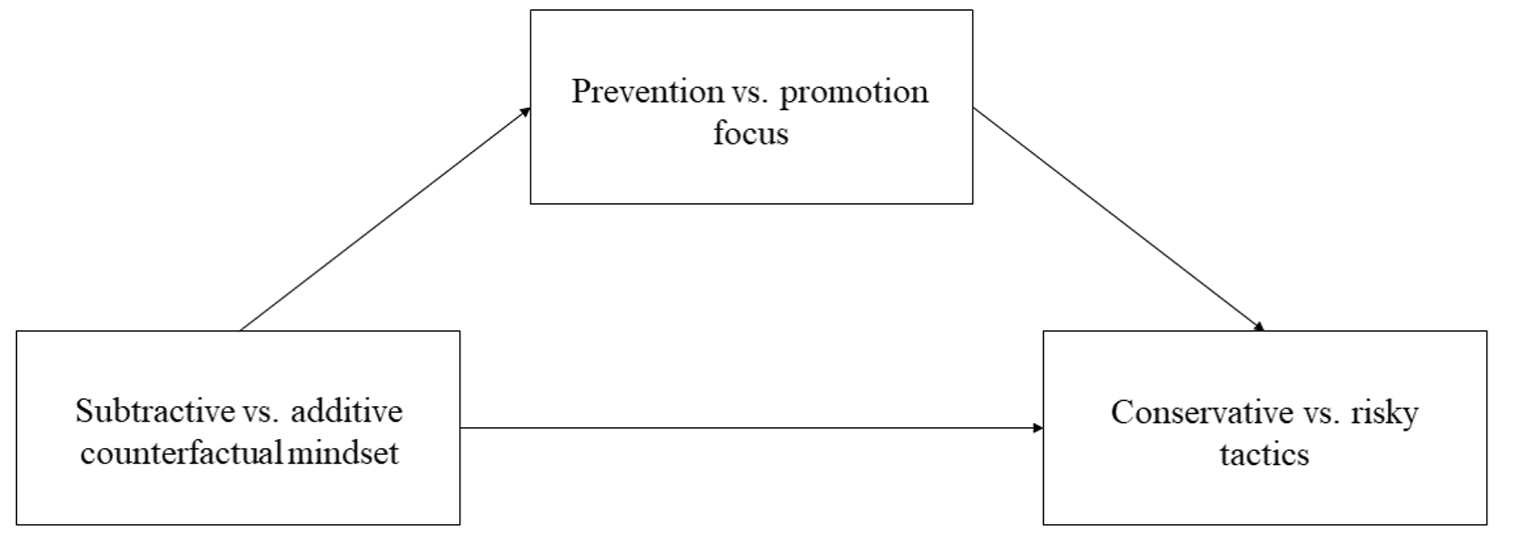
Theoretical model tested in the current research

The current research set out to extend the small body of literature on the motivational consequences of counterfactual thinking. To this end, we tested the impact of subtractive and additive counterfactual mindsets on conservative and risky tactics in decision-making. Beyond testing our main hypothesis, our studies were also suited to shed light on the underlying assumptions derived from the literature on the links between (1) counterfactual mindsets and regulatory focus, and (2) regulatory focus and behavior (see Fig. 1). We conducted four preregistered studies to examine these three relationships.

Testing our main hypothesis in Studies 1 and 2, we induced a counterfactual mindset (subtractive vs. additive vs. control) and then assessed tactics in a recognition task. To test the association between chronic regulatory focus and tactics, we conducted another preregistered correlational study (Study 3). In Study 4, we let participants generate counterfactual thoughts in response to the COVID-19 pandemic and measured their generally preferred self-regulatory strategies afterwards (as a measure of regulatory focus). In order to draw conclusions about the direction of potential effects, we implemented a neutral control condition in all studies manipulating a counterfactual mindset, thereby, addressing a limitation of previous research that did not include such a baseline (Roese et al., 1999).

Across all studies and according to our preregistrations, we included only native speakers, no psychology students or psychologists, and only participants who indicated to have seriously answered all questions. We identified statistical outliers based on studentized deleted residuals (SDR) from a regression of the main dependent variable on the main independent variable. Participants with an absolute SDR > 2.69 were excluded (see Neter et al., 1996).

In addition to the planned null hypothesis tests, we conducted exploratory Bayesian analyses with JASP (Version 0.15.0.0) for each test to see how strongly the data speak in favor of the respective H_0_ or H_1_. We followed the classification of Raftery (1995; see also Wagenmakers 2007) in interpreting the evidence in favor of either H_0_ or H_1_. For null hypothesis tests comparing only two of the three experimental conditions (e.g., subtractive vs. additive counterfactuals), the reported Bayes Factor refers to the focal contrast in a Bayesian linear regression analysis including both focal and residual contrast. We consistently report Bayes Factors indicating the likelihood of the data under the H_1_ compared to the H_0_ (i.e., BF_10_). Dividing 1 by the respective value, one arrives at the likelihood of the data under H_0_ compared to H_1_.

## Study 1

Study 1 served as a first test of our prediction that a subtractive counterfactual mindset should lead to more conservative tactics in a subsequent unrelated task, whereas an additive counterfactual mindset should increase the use of risky tactics.

## Method

### Participants

A total of 258 UK citizens recruited via Prolific Academic completed our preregistered (see https://aspredicted.org/bc7ke.pdf) online experiment. Based on our preregistered criteria, we excluded 15 participants. In addition, 12 participants were excluded because they failed to generate any counterfactual thoughts in the additive or subtractive condition. Including these participants did not change results. This left a final sample of *N* = 231 (152 women, age: *M* = 36.75 years, *SD* = 14.15, range = 18–70). A sensitivity analysis revealed that with a sample of this size we would be able to detect a small-to-medium effect (*f* = 0.21) in a one-way Analysis of Variance (ANOVA) with three groups assuming α = 0.05 and (1-β) = 0.80. Participants received £1.00 for their participation.

### Procedures

Participants were randomly assigned to one of three experimental conditions (counterfactual mindset: subtractive vs. additive vs. control). At the outset of the study, we presented the target words for the recognition memory task that was applied later as a measure of tactics that has been shown to be influenced by regulatory focus (e.g., Crowe & Higgins 1997). Participants were, however, unaware of the following recognition task and we did not instruct them to memorize the words. Rather we told them that they would read a couple of invented words that would be used for future research and that this was unrelated to the rest of the study. The presentation of the target words was closely adapted to Crowe & Higgins (1997). The stimuli consisted of 20 nonsense words that were presented in a randomized order for two seconds each before the next word appeared automatically on the screen. Each word contained five letters with vowels at the second and fourth position and consonants at the first, third, and fifth position (e.g., BUVAL, MOTUK, SEMIP). Participants subsequently answered four questions on the invented words (e.g., how complicated they were). These were, however, irrelevant to the current research but only included in order to keep up the cover story. To increase the time delay between the encoding of the target words and the recognition task, we let participants answer some questionnaires asking for their sociopolitical attitudes as well as for how they are dealing with everyday situations.

Next, the counterfactual mindset manipulation followed (adapted from Markman et al., 2007). Participants in the two counterfactual conditions were asked to remember a negative event that happened to them during the last year. Then they were told that after such events, people often have thoughts like “If only I had NOT…” (in the subtractive condition) or “If only I had…” (in the additive condition). On the subsequent page, participants were asked to list as many thoughts as came to their mind, how they could have improved the outcome of the remembered event. They had a maximum of three minutes to generate either subtractive (completing the sentence “If only I had not…the outcome would have been better”) or additive thoughts (completing the sentence “If only I had…the outcome would have been better”). Participants in the neutral control condition were not asked to remember any negative event but directly moved to the next section.

Directly after the counterfactual mindset manipulation, we presented the recognition task to participants. We instructed them that they would now read a sequence of invented words and that some of these words had been presented to them earlier. They would have to decide for each word, whether they had seen it before in this study or not – clicking on either a “yes”- or a “no”-button in a forced choice paradigm. A sequence of 40 words was presented to participants in a randomized order, 20 of them stemming from the initial encoding phase and 20 of them being new but constructed in the same way. Each word remained on the screen until participants decided on whether they had seen it before or not.

After having completed all 40 trials in the recognition task, we assessed participants’ affect in response to remembering the negative event and the experienced difficulty of generating counterfactuals (both only in the two counterfactuals conditions). In addition, we let participants rate some exploratory questionnaires not relevant to the current research. Finally, they were asked for their demographic data, thanked, and debriefed.

### Measures

*Conservative or risky tactics.* The task used to measure tactics in decision-making allows for distinguishing two kinds of response biases: conservative or risky. According to Crowe & Higgins (1997), leaning towards “no” responses in a memory recognition task represents a conservative bias, whereas tending towards “yes” responses indicates a risky bias. To calculate the indicator of response bias, we relied on the procedure used in previous research on self-regulation and decision-making (Crowe & Higgins, 1997) as well as more general literature on signal detection theory (Macmillan & Creelman, 2005; Stanislaw & Todorov, 1999). The *β* criterion indicates participants tendency to respond “yes” or “no” in the recognition task and is calculated with the formula:

$$\beta =exp\left\{\frac{{probit\left(F\right)}^{2}-{probit\left(H\right)}^{2}}{2}\right\}$$

F stands for the false-alarm rate (i.e., number of false alarms divided by the total number of noise trials) and H for the hit rate (i.e., number of hits divided by the total number of signal trials). A completely unbiased response pattern is represented by *β* = 1. Values of *β* > 1 indicate a conservative bias (i.e., toward responding “no”), whereas values of *β* < 1 are associated with a risky bias (i.e., toward responding “yes”). Thus, higher values of *β* signify more conservative decision-making.

*Affect.* We used five bipolar items (e.g., 1 = *depressed* to 9 = *elated*) to ask participants (only in the counterfactual mindset conditions) how remembering the negative event made them feel right now (Roese, 1994; α = 0.88).

*Difficulty.* Participants in the counterfactual conditions were also asked how difficult they found it to come up with thoughts in response to remembering the negative event. The two administered items (“It was easy for me to come up with ideas how I could have improved the outcome of the event”, “I had difficulties to think about concrete behaviors that could have improved the event”; 1 = *not at all* to *7* = *very*) were subsumed to one scale after recoding the first item, *r*(141) = 0.69, *p* < .001. Thus, higher scores represented higher experienced difficulty.

## Results

**Manipulation check.** As was done in previous research using counterfactual mindset manipulations (Roese, 1994), we calculated a manipulation check score for participants in the additive and subtractive counterfactual condition. We built this score by subtracting the number of additive counterfactuals from the number of subtractive counterfactuals generated by each participant. Thus, higher scores represent a higher frequency of subtractive counterfactuals. An independent two-sample *t* test confirmed that participants in the subtractive condition generated subtractive counterfactuals more frequently than those in the additive condition, *t*(139) = 10.07, *p* < .001, *d* = 1.70, 95% CI [1.31, 2.08], BF_10_ > 150 (for Means and Standard Deviations, see Table 1).

**Table 1:** Means (Standard Deviations) across conditions and correlations between measures (Study 1).

	Subtractive counterfactuals (*n* = 73)	Additive counterfactuals (*n* = 68)	Control condition (*n* = 90)	(1)	(2)	(3)	(4)
(1) Manipulation check	1.21 (2.20)	-2.02 (1.50)	-	-	− 0.11	0.02	− 0.001
(2) Tactics	1.03 (0.58)	1.14 (0.74)	0.98 (0.38)		-	− 0.07	− 0.05
(3) Affect	4.03 (1.39)	3.82 (1.60)	-			-	0.22*
(4) Difficulty	3.30 (1.66)	3.15 (1.53)	-				-

*p < .05, **p < .01, ***p < .001

**Conservative or risky tactics.** We tested our hypothesis that subtractive counterfactuals would increase the use of conservative tactics compared to additive counterfactuals with a one-way ANOVA using *β* as the dependent variable. There was no evidence for differences between conditions, *F*(2, 228) = 1.49, *p* = .227, η² = 0.01, 90% CI [0.00, 0.04]^1^, BF_10_ = 0.17. More central to our prediction, the planned contrast comparing the subtractive and additive condition (+ 1 subtractive, -1 additive, 0 control) was not significant, *t*(228) = -1.11, *p* = .270, *d* = -0.15, 95% CI [-0.48, 0.18], BF_10_ = 0.25. Thus, there was no evidence that subtractive and additive condition led to different response patterns in the recognition task. The Bayes factor suggests that the observed data is about four times more likely under the H_0_ than under the H_1_. Furthermore, there was no evidence that subtractive and additive condition taken together differed from the neutral control condition, *t*(228) = -1.35, *p* = .178, *d* = -0.18, 95% CI [-0.44, 0.09], BF_10_ = 0.33 (residual contrast: -1 subtractive, -1 additive, + 2 control).

**Exploratory analyses.** An independent two-samples *t* test revealed no evidence regarding *affect* induced by either subtractive or additive counterfactual thoughts, *t*(139) = 0.84, *p* = .400, *d* = 0.14, 95% CI [-0.19, 0.47], BF_10_ = 0.25. Likewise, there was no evidence that the task of generating either subtractive or additive counterfactual thoughts was judged as more or less *difficult*, *t*(139) = 0.55, *p* = .586, *d* = 0.09, 95% CI [-0.24, 0.42], BF_10_ = 0.21.

## Discussion

The results obtained in Study 1 provide no support for our prediction that a subtractive (additive) counterfactual mindset would lead to the use of conservative (risky) tactics in decision-making. Different from what we hypothesized, subtractive and additive counterfactual mindsets did not lead to the use of different response styles in a recognition task. More precisely, neither did a subtractive counterfactual mindset foster conservative tactics, nor did an additive counterfactual mindset trigger risky tactics. One reason for this unexpected pattern of results might lie in the instructions we used to establish a counterfactual mindset. In both additive and subtractive condition, the example given in the instructions highlighted the possibility of avoiding a negative outcome (i.e., not getting seriously injured) instead of pointing to achieving a positive outcome. Thus, the instructions might not have been neutral with regard to regulatory focus. This could also explain why in both counterfactual conditions, the means descriptively pointed into the direction of a conservative bias. The exploratory analyses yielded no evidence for differences between the two mindset types with regard to both affect and perceived difficulty.

## Study 2

In Study 2, we sought to rule out some potential limitations of Study 1 and to gain more insights on the motivational effects of counterfactual mindsets. To account for the former, we chose the counterfactuals manipulation of Roese and colleagues (1999) which was explicitly created to be neutral with regard to regulatory focus and which is the study we mainly based our predictions on. In addition to assessing actual behavior, we measured participants’ self-reported willingness to perform risky behaviors as a secondary indicator of tactics. This time we also aimed to shed light on the presumably underlying motivational states by measuring situational regulatory focus. In doing so, Study 2 allowed us to not only test our main prediction, but also to explore the validity of the underlying assumption that subtractive and additive counterfactual mindsets would differentially affect regulatory focus.

## Method

### Participants

In order to safeguard against potential statistical power issues, we aimed at recruiting 100 participants per condition. We recruited 302 participants via Prolific Academic. Twenty-two participants were excluded based on our preregistered criteria (see https://aspredicted.org/pu7hr.pdf) and another five for failing to generate any counterfactual thoughts, while results did not differ when including them. Our final sample consisted of *N* = 275 (184 women, *M*_age_ = 37.16 years, *SD* = 12.24, range = 18–74). With this sample size we would be able to detect a small-to-medium effect (*f* = 0.19) in a one-way ANOVA with three groups assuming α = 0.05 and (1-β) = 0.80. Participants were paid £1.00 for their participation.

### Procedures

Study 2 was an online experiment with the same three conditions as before (counterfactual mindset: subtractive vs. additive vs. control). The procedure was closely adapted to that of Study 1. First, the encoding phase of the recognition task took place as in Study 1. Second, to manipulate the counterfactual mindset, participants were asked to write down some details about the negative event they remembered (Roese, 1994; Roese et al., 1999). Then, they had three minutes to provide up to five examples of how they could have improved the outcome of the remembered event. As before, participants in the subtractive condition were prompted to indicate what they “should not have done”, whereas those in the additive condition responded to the sentence “What I should have done”. This time, also participants in the control condition were asked to remember a negative event and to provide some information about it, but without soliciting any counterfactuals. They were asked to indicate how long the event was ago, whether others were involved, and how often they think about this event.

After the counterfactual manipulation, we measured situational regulatory focus, risky behavioral intentions, affect elicited by remembering the negative event, and the perceived difficulty of generating counterfactuals (the latter only in the subtractive and additive condition). Then, participants responded to the same recognition task as in Study 1. Finally, demographic data was retrieved.

### Measures

*Situational regulatory focus.* To assess situational promotion (“To what extent are you going to focus on avoiding negative outcomes in the future?”) and prevention focus (“To what extent are you going to focus on achieving positive outcomes in the future?”), we used one item each (Gino & Margolis, 2011; 1 = *not at all* to 7 = *very much*). The items were positively correlated (see Table 2).

**Table 2:** Means (Standard Deviations) across conditions and correlations of all measures (Study 2)

	Subtractive counterfactuals (*n* = 93)	Additive counterfactuals (*n* = 85)	Control condition (*n* = 97)	(1)	(2)	(3)	(4)	(5)	(6)	(7)
(1) Manipulation check	1.93 (2.58)	-3.24 (1.69)	-	-	− 0.07	0.01	− 0.04	0.11	0.02	0.16*
(2) Tactics	0.92 (0.38)	1.01 (0.49)	0.96 (0.45)		-	− 0.05	− 0.16**	− 0.05	− 0.03	0.03
(3) Risky behavioral intentions	2.45 (0.94)	2.51 (0.89)	2.36 (1.00)			-	− 0.01	− 0.01	− 0.05	0.02
(4) Situational prevention focus	5.08 (1.64)	4.92 (1.50)	5.02 (1.68)				-	0.25***	− 0.17**	− 0.13
(5) Situational promotion focus	5.98 (1.10)	5.82 (0.94)	5.96 (1.09)					-	− 0.02	0.05
(6) Affect	3.56 (1.78)	3.26 (1.50)	3.44 (1.60)						-	− 0.16*
(7) Difficulty	4.15 (1.57)	3.72 (1.57)	-							-

*p < .05, **p < .01, ***p < .001

*Risky behavioral intentions* were measured with a scale adapted from previous research in which these intentions were enhanced after a situational induction of promotion (vs. prevention) focus (Gino & Margolis, 2011). The scale consisted of five items that asked for the likelihood with which participants would perform a certain risky action right now (e.g., “Cheating on an exam”; 1 = *extremely unlikely* to 5 = *extremely likely*) and showed good internal consistency (α = 0.81).

*Affect.* We used the same measure of affect as in Study 1 (α = 0.91). This time, however, all participants responded to these items, because participants in the control condition also remembered a negative event. Again, higher values signify more positive affect.

*Difficulty.* The same two items as in Study 1 were used to measure the perceived difficulty of generating counterfactuals, *r*(178) = 0.48, *p* < .001.

*Conservative or risky tactics.* The preference for using conservative or risky tactics in the recognition task was assessed as in Study 1.

## Results

**Manipulation check.** We used the same manipulation check score as in Study 1 comparing the frequency of generated subtractive and additive counterfactuals. Participants in the subtractive condition listed subtractive counterfactuals with higher frequency than participants in the additive condition, *t*(176) = 15.61, *p* < .001, *d* = 2.35, 95% CI [1.97, 2.73], BF_10_ > 150 (for Means and Standard Deviations, see Table 2).

**Conservative or risky tactics.** As before we calculated a one-way ANOVA with planned contrasts to test our hypothesis that subtractive counterfactuals lead to more conservative decision-making as compared to additive counterfactuals. The ANOVA did not reveal any significant differences between experimental conditions, *F*(2, 272) = 0.83, *p* = .436, η² = 0.01, 90% CI [0.00, 0.03], BF_10_ = 0.08. More important to our hypothesis, there was no evidence for a difference between the subtractive and additive condition, *t*(272) = -1.29, *p* = .199, *d* = -0.21, 95% CI [-0.50, 0.09], BF_10_ = 0.29. The observed data are more than three times more likely under the H_0_ compared to under the H_1_. If anything, there was an unexpected tendency of subtractive counterfactuals resulting in a bias toward risky responses compared with an absolutely unbiased response pattern (i.e., β = 1), *t*(92) = -1.95, *p* = .055, *M*_diff_ = -0.08, 95% CI [-0.16, 0.002], BF_10_ = 0.70. There was no evidence that the neutral control condition differed from the other two conditions, *t*(272) = -0.11, *p* = .911, *d* = -0.01, 95% CI [-0.26, 0.24], BF_10_ = 0.13.

Also, for our second indicator of tactics, that is *risky behavioral intentions*, there was no evidence for a difference between the subtractive and the additive condition, *t*(176) = -0.42, *p* = .677, *d* = -0.07, 95% CI [-0.36, 0.23], BF_10_ = 0.14.

**Exploratory analyses.** We tested whether counterfactual mindsets influenced *situational regulatory focus* in line with previous research (Roese et al., 1999). Results of two independent two-samples *t* tests revealed no evidence that subtractive and additive counterfactual mindsets led to differences in situational promotion focus, *t*(176) = 1.00, *p* = .317, *d* = 0.16, 95% CI [-0.14, 0.45], BF_10_ = 0.21, or to differences in situational prevention focus, *t*(176) = 0.67, *p* = .505, *d* = 0.10, 95% CI [-0.19, 0.40], BF_10_ = 0.16.

A one-way ANOVA comparing all three experimental conditions provided no evidence for differences regarding the *affect* that resulted from remembering a negative event, *F*(2, 272) = 0.73, *p* = .481, η² = 0.01, 90% CI [0.00, 0.02], BF_10_ = 0.08. Again, we also tested whether coming up with subtractive or additive counterfactuals was perceived as more *difficult*. An independent two-samples *t* test revealed a marginal difference between conditions, *t*(176) = 1.84, *p* = .068, *d* = 0.27, 95% CI [-0.02, 0.57], BF_10_ = 0.77. Participants found it somewhat more difficult to generate subtractive as compared to additive counterfactuals.

## Discussion

In sum, the results of Study 2 bolster those of Study 1. Subtractive and additive counterfactual mindsets did not lead to different motivational outcomes. Instead, conservative or risky tactics in the recognition task as well as risky behavioral intentions remained unaffected by our manipulation and thus, we found no support for our main hypothesis. Descriptively, a subtractive counterfactual mindset even increased risky responses although the deviation from a perfectly unbiased response pattern was very small. In addition, our exploratory analyses revealed no evidence for differences in terms of self-reported regulatory focus. The exploratory results further do not indicate differences in affect as a result of our manipulation or perceived difficulty of subtractive compared to additive counterfactuals. Taken together, the results of Studies 1 and 2 suggest that the data are almost three times more likely under the H_0_ compared to under the H_1_ (aggregated BF_10_ = 0.37 for the response bias).

Besides repeatedly showing that counterfactual mindsets do not affect the use of conservative or risky tactics in a recognition task, we likewise found no effect of counterfactual mindsets on risky behavioral intentions. Both of these measures have been affected by situational inductions of regulatory focus in previous research (Crowe & Higgins, 1997; Gino & Margolis, 2011). Thus, the question arises whether the repeated null effects stem from a failure to induce the assumed motivational states via counterfactual mindsets. The exploratory results on situational regulatory focus in this study, support this interpretation. At the same time, it should be noted that the measure we used (and which was previously used as a manipulation check for induced regulatory focus; Gino & Margolis 2011) might not be ideal to solely capture regulatory focus as it conflates it with approach and avoidance tendencies (two related but arguably distinct constructs; Summerville & Roese 2008).

Another potential explanation for the outcome is that the induced motivational states do not translate into different tactics at the behavioral level. If this was the case, then a lack of an effect of counterfactual mindsets on conservative or risky tactics would not be indicative of whether counterfactual mindsets induced a certain regulatory focus. The missing or counterintuitive (higher situational prevention focus was related to less conservative tactics; see Table 2) correlations between situational regulatory focus and tactics in this study point into this direction but needs further validation due to the suboptimal measurement of regulatory focus. To validate the relationship between regulatory focus and conservative or risky tactics that we assumed based on the literature (e.g., Crowe & Higgins 1997), we conducted another correlational study.

## Study 3

The goal of Study 3 was to validate whether our measures of conservative or risky tactics were indicative of differences in regulatory focus. This relationship is suggested by previous research that reported effects of a situational regulatory focus induction on the tactics used in said recognition task (Crowe & Higgins, 1997; Friedman & Förster, 2001) and on risky behavioral intentions (Gino & Margolis, 2011), but a more complex relationship between regulatory focus and tactics has been proposed since (e.g., Scholer et al., 2010; Zou et al., 2014). To extend these findings and to validate the underlying assumption, we aimed to demonstrate a relationship between chronic prevention (promotion) focus and conservative (risky) tactics. If such a relationship could be found, this would bolster the interpretation that the counterfactual mindset manipulations in Studies 1 and 2 did not induce different regulatory foci (contrary to the findings of Roese et al., 1999). However, if such a relationship was missing, this would cast further doubt on the link between regulatory focus and the use of conservative and risky tactics – even under neutral conditions for which this assumption has been sustained (Scholer et al., 2008).

## Method

### Participants

One-hundred participants completed our online questionnaire which was distributed via Prolific Academic. Based on our preregistration (see https://aspredicted.org/xh282.pdf), we excluded seven participants, but including them did not alter the results. Our final sample consisted of *N* = 93 UK adults (39 women, age: *M* = 30.82 years, *SD* = 10.19, range: 18–66).

### Procedures and measures

The design of the study was correlational. First of all, as in Studies 1 and 2, we presented the target words for the recognition task participants were unaware of. This was followed by some questions on the presented words that were irrelevant for our research, but only served to keep up the cover story (i.e., that this part was an initial evaluation of study material). Then, participants proceeded to the “actual study” which contained the measures of interest.

First, we presented the regulatory focus scale developed by Sassenberg et al. (2012) which used a 7-point scale (1 = *does not apply at all* to 7 = *does fully apply*). The prevention scale consisted of eight items (e.g., “I am literally always following rules and regulations”, α = 0.55), the promotion scale of twelve items (e.g., “Success sets me at ease”, α = 0.84).

Second, the Regulatory Focus Questionnaire (Higgins et al., 2001) was presented on a 5-point scale. Note that we sticked to the original phrasing of the statements and response options so that not all items had the same verbal scale anchors. The prevention scale consisted of five items (e.g., “Not being careful enough has gotten me into trouble at times” from 1 = *never or seldom* to 3 = *sometimes* to 5 = *very often*, reverse item, α = 0.83), the promotion scale of six items (e.g., “I feel like I have made progress toward being successful in my life” from 1 = *certainly false* to 5 = *certainly true*, α = 0.65). We also assessed *risky behavioral intentions* with the same five items (α = 0.78) as in Study 2. Finally, participants’ recognition performance was probed.

## Results

The correlations between all measures as well as *M*s and *SD*s are presented in Table 3. Most importantly, we did not find evidence for a relationship between either measure of regulatory focus and the tactics used in the recognition task. Rather the data was at least 2.5 times more likely under the H_0_ compared to under the H_1_, all BFs_10_ < 0.40. Further validating the absence of a relationship, there was no evidence for a relationship between risky behavioral intentions and three of the four scales used to measure chronic regulatory focus all BFs_10_ < 0.52. Only a negative relationship between chronic prevention focus (on the scale from Sassenberg et al., 2012) and risky behavioral intentions was present, BF_10_ = 1.89. Of the different measures of regulatory focus, only the two promotion (but not the prevention) scales were positively correlated, BF_10_ > 150. Moreover, no evidence for a relationship between risky behavioral intentions and conservative or risky tactics in the recognition task was found, BF_10_ = 0.14.

**Table 3:** Ms and SDs as well as correlations between measures used in Study 3 (N = 93)

	*M*	SD	(1)	(2)	(3)	(4)	(5)	(6)
(1) Prevention (Sassenberg et al., 2012)	4.91	0.66	-	0.26*	0.14	0.11	− 0.24*	0.16
(2) Promotion (Sassenberg et al., 2012)	4.89	0.82		-	− 0.19	0.60***	0.04	− 0.10
(3) Prevention (Higgins et al., 2001)	3.28	0.87			-	− 0.02	− 0.17	− 0.07
(4) Promotion (Higgins et al., 2001)	3.26	0.61				-	0.001	− 0.06
(5) Risky behavioral intentions	2.87	0.96					-	0.03
(6) Tactics	1.16	0.96						-

*p < .05, **p < .01, ***p < .001

## Discussion

Other than expected, we did not find evidence for a relationship between chronic regulatory focus and the tactics used in the recognition task. That is, whether participants adopted conservative or risky tactics in decision-making was independent of their chronic regulatory focus, which we assessed with two established measures. The only relationship that was in line with the underlying assumption was the negative correlation between chronic prevention focus and risky behavioral intentions that occurred for one of the two prevention scales (notably, the one with unsatisfying internal consistency). Thus, if anything there was only weak evidence for a relationship between regulatory focus and (self-reported) conservative or risky tactics. These findings run counter previous work that found a straightforward link between prevention (promotion) focus and conservative (risky) tactics (e.g., Crowe & Higgins 1997), but ties in with more recent developments in regulatory focus research that propose a more complex relationship between general motivational orientations and actual behavior (e.g., Scholer et al., 2008).

## Study 4

Our final study was designed to test the second assumption that we derived our main hypothesis from, namely that counterfactual mindsets would affect regulatory focus. To this end, we measured the importance participants attributed to certain self-regulatory strategies, which comes very close to previous research (Roese et al., 1999). In Study 4, we made another important change to the previous studies with regard to the counterfactual mindset manipulation. Other than in Studies 1 and 2, we did not let participants freely choose which negative event they remembered. Instead, we asked participants for potential alternative behaviors in response to the recently spread COVID-19 pandemic, which was a salient event during the time the study was conducted.

## Method

### Participants

In total, 225 participants completed our study. Eight of them were excluded based on our preregistered (see https://aspredicted.org/te3fr.pdf) criteria, plus 13 participants who did not enter any counterfactual thoughts. Including these participants into the analyses did not alter results. The final sample consisted of *N* = 204 participants (149 women, age: *M* = 37.32 years, *SD* = 12.96, range: 18–75) who were UK citizens recruited via Prolific Academic. This sample size would allow us to find a small-to-medium effect (*f* = 0.22) in a one-way ANOVA with (1-β) = 0.80 at α = 0.05. Participation was remunerated with £1.00.

### Procedures

Study 4 was another online experiment with three randomly assigned conditions (counterfactual mindset: subtractive vs. additive vs. control) that were varied between subjects. At the outset of the study, we assessed participants’ chronic regulatory focus to validate that our dependent measure indeed captured differences in this motivational state. Then, we carried out the manipulation of counterfactual mindset. The procedure was similar to that of the previous studies. This time, however, we asked participants to think about their personal experiences with the situation revolving around the newly spread coronavirus, instead of a freely chosen negative personal event. Given that the study was conducted in the early stages of the COVID-19 pandemic, we asked participants whether they had heard of the outbreak of the virus and whether they had been infected. The task instructions were the same as in Study 2, relying on the manipulation of Roese and colleagues (1999). In the subtractive condition, we asked participants what they *should not* have done in face of the coronavirus. Participants in the additive condition had to give examples on what they *should* have done in face of the coronavirus. In the neutral control condition, some general questions about the experience with the pandemic were asked: when they first learned about the coronavirus, whether they know others who are directly affected by the coronavirus, and how often they think about the coronavirus and its consequences.

Directly afterwards, we measured self-regulatory strategies which was our main dependent variable. As before, we also assessed participants’ affect in response to the manipulation and the perceived difficulty of generating counterfactuals in the respective conditions. A basic demographic questionnaire followed.

### Measures

*Chronic regulatory focus* was measured with the prevention (α = 0.53) and promotion (α = 0.87) focus scale from Sassenberg et al. (2012) as in Study 3.

*Self-regulatory strategies.* We used an established scale from the regulatory focus literature in order to assess self-regulatory strategies (Hamstra et al., 2014; Sassenberg et al., 2007). Participants responded to five bipolar items that asked for actions and strategies they personally considered important (“What is most important to you?”; e.g., 1 = *taking risks* to 9 = *acting cautiously*). Importantly, other than the situational regulatory focus measure used in Study 2, this measure does not confound regulatory focus with approach and avoidance tendencies. After recoding two items, higher scores of the scale represented more vigilance. Internal consistency of the scale was rather low (α = 0.57)^2^.

*Affect.* The same scale as in the previous studies was used to measure affect (α = 0.88).

*Difficulty.* Perceived difficulty of generating counterfactuals was assessed via one bipolar item (“Thinking back, how easy or difficult did you experience the task of listing ‘if only’ thoughts regarding the coronavirus?”; 1 = *very easy* to 9 = *very difficult*).

## Results

**Manipulation check.** Based on the same score as in the previous studies, we compared the frequency of generated subtractive or additive counterfactuals between conditions. An independent two-samples *t* test proved that participants generated a higher frequency of subtractive counterfactuals in the subtractive as compared to the additive condition, *t*(131) = 12.47, *p* < .001, *d* = 2.16, 95% CI [1.74, 2.59], BF_10_ > 150 (for Means and Standard Deviations, see Table 4).

**Table 4:** Means (Standard Deviations) across conditions and correlations between measures (Study 4)

	Subtractive counterfactuals (*n* = 69)	Additive counterfactuals (*n* = 64)	Control condition (*n* = 71)	(1)	(2)	(3)	(4)	(5)	(6)
(1) Chronic prevention focus	4.85 (0.74)	5.01 (0.61)	4.84 (0.53)	-	0.33***	− 0.01	0.27***	− 0.03	0.06
(2) Chronic promotion focus	5.08 (0.91)	4.94 (0.72)	4.62 (0.80)		-	0.09	− 0.41***	0.16*	0.01
(3) Manipulation check	1.46 (2.56)	-3.25 (1.67)	-			-	− 0.07	0.02	0.08
(4) Self-regulatory strategies	5.51 (1.13)	5.73 (1.19)	5.67 (1.06)				-	− 0.20**	− 0.06
(5) Affect	4.01 (1.50)	3.98 (1.43)	3.41 (1.13)					-	0.30***
(6) Difficulty	6.25 (2.19)	5.72 (2.20)	-						-

*p < .05, **p < .01, ***p < .001

**Self-regulatory strategies.** Testing whether a subtractive (vs. additive) counterfactual mindset would increase the importance attributed to prevention (over promotion) strategies, we ran a one-way ANOVA. No evidence for differences between conditions were found, *F*(2, 201) = 0.70, *p* = .496, η² = 0.01, 90% CI [0.00, 0.03], BF_10_ = 0.09. Against our prediction and contrary to previous research, there was no evidence for subtractive counterfactuals leading to a preference for prevention strategies compared to additive counterfactuals, *t*(201) = -1.14, *p* = .258, *d* = -0.19, 95% CI [-0.53, 0.15], BF_10_ = 0.28. The observed data were 3.5 times more likely under the H_0_ than under the H_1_. Likewise, there was no evidence for a difference between the neutral control condition and the other two conditions, *t*(201) = 0.32, *p* = .750, *d* = 0.04, 95% CI [-0.24, 0.33], BF_10_ = 0.16.

Validating that our dependent measure reflected chronic differences in regulatory focus, we found a significant negative relationship between self-regulatory strategies (with higher scores representing more vigilance) and chronic promotion focus as well as a significant positive relationship with chronic prevention focus, both BFs_10_ > 150 (see Table 4).

**Exploratory analyses.** Exploring effects of counterfactual mindsets on *affect* with a one-way ANOVA, we found differences between experimental conditions, *F*(2, 201) = 4.26, *p* = .015, η² =, 90% CI [0.004, 0.09], BF_10_ = 2.11. Contrast analyses revealed no evidence that affect differed between subtractive and additive counterfactuals, *t*(201) = 0.10, *p* = .918, *d* = 0.02, 95% CI [-0.32, 0.36], BF_10_ = 0.15. However, both types of counterfactuals improved affect as compared to the neutral control condition, *t*(201) = -2.91, *p* = .004, *d* = -0.44, 95% CI [-0.73, -0.14], BF_10_ = 7.91. With regard to the perceived *difficulty* of generating counterfactuals, no evidence for a difference between subtractive and additive counterfactuals was found in a two-samples *t* test, *t*(131) = 1.38, *p* = .169, *d* = 0.12, 95% CI [-0.22, 0.47], BF_10_ = 0.44.

## Discussion

The results of Study 4 call into question that a subtractive (additive) counterfactual mindset would lead to a preference for prevention over promotion strategies. This is in contrast to the results of previous research linking counterfactual structure to regulatory focus (Roese et al., 1999). Thus, the failure to find an effect of counterfactual mindsets on tactics in the previous studies might not only be due to regulatory focus not translating into the respective tactics (as suggested by Study 3), but similarly be due to difficulties inducing the assumed motivational states via counterfactual mindsets. These results have broader implications for the literature on counterfactual thinking and regulatory focus that will be discussed below.

It is noteworthy that the mindset induction in this study referred to an event (i.e., the COVID-19 pandemic) that affected every part of people’s lives at that time and should, therefore, be a much more involving manipulation compared to the one used in Studies 1 and 2. The fact that such a strong situational factor and the counterfactual thoughts that arise in response to it (which probably happens frequently in people’s everyday lives) does not affect people’s motivation in terms of regulatory focus is in itself a relevant insight when thinking, for instance, about people’s motivation to engage in health protecting behaviors or to follow official health guidelines (which are likely to be prevention focused). Based on the current findings one would assume that these behaviors are not affected by counterfactual thoughts about one’s behavior in the pandemic.

An interesting pattern emerged with regard to the affective consequences of counterfactual mindsets. Compared to a neutral control condition, generating (any) counterfactuals in response to one’s personal experiences with the coronavirus led to more positive affect. Given the exploratory character of this analysis and the fact that no similar pattern was found in the previous studies, this result should, however, certainly not be overstated.

## General discussion

The aim of the current research was to examine the motivational consequences of counterfactual mindsets and to complement the small body of research conducted in this area. We tested (1) our main hypothesis that a subtractive (additive) counterfactual mindset would lead to the adoption of conservative (risky) tactics as well as the underlying assumptions that (2) a subtractive (additive) counterfactual mindset would foster a prevention (promotion) focus, and that (3) prevention (promotion) focus was in turn related to the adoption of conservative (risky) tactics. Across four preregistered studies using different established measures and manipulations of the core concepts, we did not find support for any of these relationships. In Studies 1 and 2, a counterfactual mindset manipulation did not affect the tactics used in a recognition task. Study 2 extended these results to self-reported risky behavioral intentions which remained unaffected as well. Study 3 and 4 served to test the underlying assumptions and found that neither chronic regulatory focus was related to the previously applied measures of tactics (Study 3) and that a counterfactual mindset manipulation did not affect regulatory focus (Study 4). First and foremost, our results indicate that counterfactual structure (i.e., subtractive vs. additive) likely has no effect on the use of conservative or risky tactics in subsequent situations. There are two possible explanations why no such an effect occurred in the first place and both of them cast doubts on links that have been long-established in the literature.

## Counterfactual structure and regulatory focus

Despite only limited direct empirical evidence (Roese et al., 1999), the assumption that subtractive (additive) counterfactuals elicit a prevention (promotion) focus has dominated the counterfactuals literature up to this point (Epstude & Roese, 2008; Roese & Epstude, 2017). Our results suggest that this might have been a premature conclusion or one that only holds under specific boundary conditions. In Study 2, we did not find an effect of the counterfactual mindset manipulation on situational regulatory focus. Although the items had been used in prior research as a manipulation check for a regulatory focus induction (Gino & Margolis, 2011), we are aware that they were conflated with approach and avoidance tendencies and, thus, do not represent an ideal measure of regulatory focus. In Study 4, however, our dependent measure did not show such a confound as both prevention and promotion items equally referred to approach or avoidance (e.g., “acting thoroughly” vs. “acting superficially” or “following rules” vs. “trying new things”). This measure likewise remained unaffected by the counterfactual mindset manipulation. Thus, a failure to find behavioral effects of counterfactual mindsets might be due to a failure to induce a certain regulatory focus via this mental procedure.

Roese et al., (1999) demonstrated a link between regulatory focus and counterfactual thinking. However, they focused on slightly different aspects of regulatory focus (i.e., predictions regarding sufficiency and necessity of goals). We looked at variables that are more closely tied to behavior. It might very well be that an autobiographical recall paradigm inducing counterfactual mindsets, may not be sufficient to elicit related personal behavior. Much of the previous research on regulatory focus examined judgments that were somewhat removed from the self, like evaluations of political decisions (Boldero & Higgins, 2011), or moral dilemmas (Cornwell & Higgins, 2013). In previous research on counterfactuals, perceived control is a variable that gained special importance. Situations in which a failure resulted from a self-initiated action (thus being controllable) is linked to a high number of counterfactuals (Roese et al., 2017). Therefore, one could assume that when specifically focusing on these types of counterfactuals, variables linked to personal behavior might be affected more strongly. Given the small body of research on the counterfactual-regulatory focus link, the lack of evidence in our studies is indicative for the need to revisit the strength of this link.

As often, the observation of null effects demands the search for potential moderators that would make the emergence of an effect more or less likely. In our case, a closer inspection of the dependent variable used in previous research might be insightful. Roese et al., (1999) asked participants to evaluate the importance of self-regulatory strategies following a counterfactual mindset manipulation. Apparently, the strategies did not only differ in the regulatory focus they comprised, but also in the degree of action or inaction that was inherent. The promotion focus items such as “making new friends”, “studying harder”, or “being more active in politics” were worded in terms of action, whereas most of the prevention focus items such as “not making enemies”, “not looking unfashionable”, or “avoiding getting fat” focused on inaction. One emergent speculation is that counterfactual mindsets might rather influence action or inaction than promotion or prevention. This interpretation is plausible given that additive (subtractive) counterfactuals result from failures of inaction (action). Thus, counterfactual mindsets might facilitate the behavioral tendency opposite to the failure’s cause, that is additive counterfactuals fostering action and subtractive counterfactuals fostering inaction. The measures we used, however, do not appear to confound regulatory focus with (in)action and, thus, might have been more adequate to test for the relationship of interest. Future research could, for instance, use dependent measures that clearly distinguish promotion/prevention from action/inaction and test whether counterfactual mindsets affect these measures to a different degree.

## Regulatory focus and conservative or risky tactics

A second reason for not finding an effect of counterfactual mindsets on tactics lies in the possibility that regulatory focus is not linked to conservative or risky tactics in a straightforward way. Although only correlational, the results of Study 3 suggest that chronic regulatory focus translates neither into the tactics used in a recognition task nor into self-reported risky behavioral intentions (the same measures that were unaffected by the counterfactual mindset manipulation in Studies 1 and 2). This finding is in contrast to previous research that found an effect of situational regulatory focus inductions on exactly these measures (Crowe & Higgins, 1997; Friedman & Förster, 2001; Gino & Margolis, 2011). At the same time, our results correspond to more recent research on regulatory focus that suggests a more complex relationship between prevention (promotion) focus and conservative (risky) tactics (e.g., Scholer et al., 2008; 2010; Zou et al., 2014).

Following these recent developments, the choice of conservative or risky tactics does not only depend on the motivational state, but also on the possibilities the behavioral options provide with regard to pursuing the underlying goal (Higgins, 2018; Scholer et al., 2019; Zou et al., 2020). There is evidence that prevention-focused individuals are likely to prefer risky over conservative tactics when they serve the purpose of turning a loss into a non-loss (Scholer et al., 2008, 2010). Likewise, promotion-focused individuals switch to conservative tactics, when their gains are at stake (Zou et al., 2014). So far, it has been theorized, however, that under conditions that do not provide clear options for goal pursuit (as in the recognition task we used), people would stick with their default tactics (Scholer et al., 2008) – that is, prevention (promotion) focus fostering conservative (risky) tactics – which could explain earlier results (Crowe & Higgins, 1997; Friedman & Förster, 2001). Our results indicate that even this relationship cannot be taken for granted and might need to be revisited.

The situations participants remembered during the counterfactual mindset induction were negative in nature and, thus, potentially involved losses leading to perceptions of being below the “status quo”. In such cases, prevention focused participants use risky tactics to pursue their vigilant strategies if this is the only possibility to return to the status quo, but not if there are conservative tactics available that serve the same purpose (Scholer et al., 2010). In our studies, the counterfactual mindset manipulation which might have caused a perceived reduction of the status quo was unrelated to the recognition task. Thus, it is unlikely that the second task offered a possibility for participants to return to the status quo by using either conservative or risky tactics. Interestingly, if no possibility to return to the status quo was offered in previous research, prevention focused participants showed no preference for either conservative or risky tactics (although there was a non-significant tendency towards preferring the risky tactic; Scholer et al., 2010; Study 4). This is consistent with our results that show no response bias after a subtractive mindset induction (but a non-significant trend towards risky behavior; Study 2). Taken together, even if a subtractive (additive) counterfactual mindset manipulation elicited prevention (promotion) focus, this motivational state might not necessarily lead to the use of more conservative (risky) tactics, which could partly explain the null results.

The current studies also have broader implications for the measurement of both regulatory focus and tactics. First, the missing correlation between the two prevention scales in Study 3 calls into question whether these two scales actually measure the same construct. Together with the suboptimal internal consistency of some of the scales, this clearly calls for developing better measures of regulatory focus (Summerville & Roese, 2008). Second, self-reported intentions do not necessarily translate into actual behavior when it comes to risk-taking as indicated by the lack of a relationship between risky behavioral intentions and the adoption of conservative or risky tactics. This highlights the necessity to include both types of measures (as in our studies) to get a complete picture when investigating the behavioral consequences of regulatory focus.

## General implications for the counterfactuals research

From a theoretical perspective, a counterfactual mindset should influence behavioral tendencies via the so-called content-neutral pathway (Epstude & Roese, 2008). Regulatory focus should exert influence via this pathway, too. The finding that two concepts do not lead to a change in behavior intentions, illustrates the need for a more systematic examination of the content-neutral pathway. The key idea is that counterfactuals may exert influence on behavior going beyond the narrowly defined problem they center on. The findings summarized by this pathway are diverse in nature. A more stringent definition and examination of the specific mechanisms is needed to clarify whether these findings can and should be seen as having similar effects on behavior.

Our studies also highlight the necessity for a closer inspection of the stability and effectiveness of counterfactual mindset inductions. While our studies are not direct replications of existing findings, they apply methods that have been used in the past. The literature suffers from a lack of manipulation checks that are suitable to test the effectiveness of the priming method. Changes in the dependent variable are seen as an indicator of the effectiveness of the priming. That makes it very difficult to interpret null effects. Future research needs to establish stricter tests of when the priming is effective and develop a set of manipulation checks that reliably demonstrate that.

The standard induction of a counterfactual mindset involves participants imagining a situation in which they almost win a prize at a lottery (Galinsky & Moskowitz, 2000). This is typically followed by a measure of performance in a problem-solving or creativity task (e.g., Kray et al., 2006). Only few studies deviated from that approach by asking participants to reflect on a more personal event (Markman et al., 2007). Similar to the latter approach, our studies also focused on more self-related thoughts. The biggest difference between our studies and the existing body of research is the sample size used. Much of the existing research used samples ranging from *N* = 7 to *N* = 15 per condition (Galinsky & Moskowitz, 2000; Kray et al., 2006). Our studies adhered more to the current standards using much larger samples. The current findings, thus, also clearly highlight the need for replication of previous studies on counterfactuals under conditions that live up to current standards in psychological research.

## Conclusion

In the current research, we set out to extend the small number of studies on the motivational consequences of counterfactual mindsets. Based on previous findings, we predicted that a subtractive (vs. additive) counterfactual mindset facilitates conservative and reduces risky tactics in decision-making. However, we did not find such an effect of counterfactual mindsets. Neither subtractive nor additive counterfactuals had an influence on the tactics participants adopted. In addition, the current research puts doubt on the assumptions that led us to predict such an effect in the first place. Notwithstanding (or just for) these null effects, the present research contributes to the literature on counterfactual thinking and motivation by questioning relationships between variables that have been long-established in the literature.

## Data Availability

.The data and code of the studies reported in this article are publicly available on PsychArchives (data: http://dx.doi.org/10.23668/psycharchives.8133, code: http://dx.doi.org/10.23668/psycharchives.8132). Study materials are available in the online Supplemental Material of this article. Links to preregistrations can be found within the methods sections of the respective studies.

## Footnotes

1 Based on the recommendations of Lakens (2014) and Steiger (2004), we report 90% CI for η². They were calculated using the SPSS script provided by Smithson (2001).

2 Removing one item (i.e., 1 = *acting thoroughly* to 9 = *acting superficially*) improved the internal consistency (α = 0.63) but did not change the results. Thus, the reported analyses refer to the complete scale which we preregistered.
